# Overexpression of the Halophytic Vacuolar Na^+^/H^+^ Antiporter Enhances Salt Tolerance in *Arabidopsis thaliana*

**DOI:** 10.3390/plants15121883

**Published:** 2026-06-17

**Authors:** Roksana Aftab, Hiromi Suzuki, Yuichi Tada

**Affiliations:** 1 Graduate School of Bionics, Tokyo University of Technology, 1404-1 Katakura, Hachioji, Tokyo 192-0982, Japan; aftab.teu26@gmail.com; 2 School of Bioscience and Biotechnology, Tokyo University of Technology, 1404-1 Katakura, Hachioji, Tokyo 192-0982, Japan; suzukihrm@stf.teu.ac.jp

**Keywords:** vacuolar Na^+^/H^+^ antiporters, *Sporobolus virginicus*, *Arabidopsis thaliana*, salt tolerance, halophytes

## Abstract

The use of genes derived from halophytes represents a promising strategy for enhancing salt tolerance. Among salt tolerance mechanisms, vacuolar Na^+^/H^+^ exchangers (NHXs) play a central role in cellular Na^+^ sequestration in both halophytes and glycophytes. In this study, we identified the *SvNHX2* gene from the halophyte *Sporobolus virginicus* and characterized its role in salinity tolerance. SvNHX2 expression was strongly induced by salt stress, particularly in shoots in *S. virginicus*. *Arabidopsis thaliana* plants overexpressing *SvNHX2* exhibited significantly improved growth at the vegetative growth stage under 100 and 125 mM NaCl on agar plates and at the reproductive growth stage under 100 mM NaCl in hydroponic systems, although no clear correlation was observed between transgene expression levels and tolerance. Ion analysis showed that SvNHX2 overexpression increased Na^+^ accumulation in roots under NaCl stress, suggesting enhanced vacuolar Na^+^ sequestration, while K^+^ levels remained comparable to the wild type. These findings indicated that SvNHX2 contributes to salt tolerance primarily by promoting Na^+^ compartmentalization, and that its capacity is comparable to those of NHX proteins in glycophytes. In this study, we provide additional insights into the function of halophytic NHX and demonstrated that *SvNHX2* is a valuable gene for engineering salt-tolerant crops.

## 1. Introduction

Soil salinity is a major abiotic stress affecting more than 20% of irrigated land worldwide, and up to 50% of arable land is projected to become salinized by 2050 [1]. To meet the food demands of a global population projected to reach 9.7 billion by 2050, agricultural production must increase by approximately 70% relative to current levels [2]. However, as most crop species are sensitive to salinity, the expansion of saline soils represents a critical threat to global food security.

Under saline conditions, Na^+^ enters root cells through ion channels, transporters, or via the apoplastic pathway [3]. Excessive cytosolic Na^+^ accumulation disrupts cellular homeostasis, causing osmotic stress, enzyme inhibition, and ionic imbalance [4]. In contrast, sequestration of Na^+^ into vacuoles contributes to osmotic adjustment by utilizing Na^+^ as an osmoticum [5]. Thus, maintaining intracellular K^+^ and Na^+^ homeostasis is fundamental to plant cell function. Vacuolar Na^+^/H^+^ antiporters (NHXs) play a central role in this process by compartmentalizing Na^+^ into vacuoles, thereby mitigating cytosolic Na^+^ toxicity and preserving K^+^/Na^+^ balance [6,7,8,9]. Overexpression of *NHX* genes from diverse plant species has been shown to enhance salt tolerance in transgenic plants. *NHX* genes have been characterized in both glycophytes and halophytes, underscoring their conserved role in salt stress adaptation as follows. In glycophytes, representative examples include *AtNHX1* from *Arabidopsis thaliana* [6,10,11,12], *TaNHX2* from wheat [13,14], *OsNHX2* from rice [15], *PvNHX1* from switchgrass (*Panicum virgatum*) [16], *StNHX1* from *Solanum torvum* [17], and *LeNHX2* from *Solanum lycopersicum* [18,19]. Similarly, several halophytic *NHX* genes have been functionally characterized in heterologous systems, including *ThNHX1* from *Thellungiella halophila* [20], *SsNHX1* from *Salsola soda* [21], *SsNHX1* from *Suaeda salsa* [22], *HcNHX1* from *Halostachys caspica* [23], *SeNHX1* from *Salicornia europaea* [24,25], *SucNHX1* from *Suaeda corniculata* [26], *BvNHX1* from sugar beet [27], and *PutNHX1* from *Puccinellia tenuiflora* [25]. Although halophytes possess sophisticated mechanisms for Na^+^ exclusion, tissue-specific distribution, and vacuolar sequestration [25,28,29], transgenic plants expressing halophytic *NHX* genes generally exhibit salt tolerance levels comparable to those expressing glycophytic *NHXs*.

*Sporobolus virginicus*, commonly known as seashore dropseed grass, is a halophytic species that inhabits saline coastal environment, maintains controlled concentrations of Na^+^ under NaCl stress, and exhibits a salinity tolerance up to 1.5 M NaCl [30,31,32], a concentration lethal to not only glycophytes but also most halophytes. Although Na^+^ content increases under salt stress in *S. virginicus*, its accumulation is tightly regulated to prevent excessive cytosolic Na^+^, whereas rice exhibits rapid Na^+^ accumulation leading to severe damage and death [32,33]. NHX transporters are key regulators of intracellular Na^+^ homeostasis; however, the properties of NHX derived from this extremely salt-tolerant halophyte and its potential application to molecular breeding for salt-tolerant plants have not been investigated.

In this study, we cloned *SvNHX2*, a gene for vacuolar Na^+^/H^+^ antiporter from *S. virginicus*, and produced transgenic *Arabidopsis* plants overexpressing *SvNHX2*. To evaluate the physiological impact of *SvNHX2* overexpression and its potential for salt-tolerance breeding, we analyzed growth performance, ion accumulation, and K^+^ and Na^+^ content in transgenic Arabidopsis lines under control and NaCl stress conditions.

## 2. Results

### 2.1. Cloning and Characterization of SvNHX2

**Figure 1:**
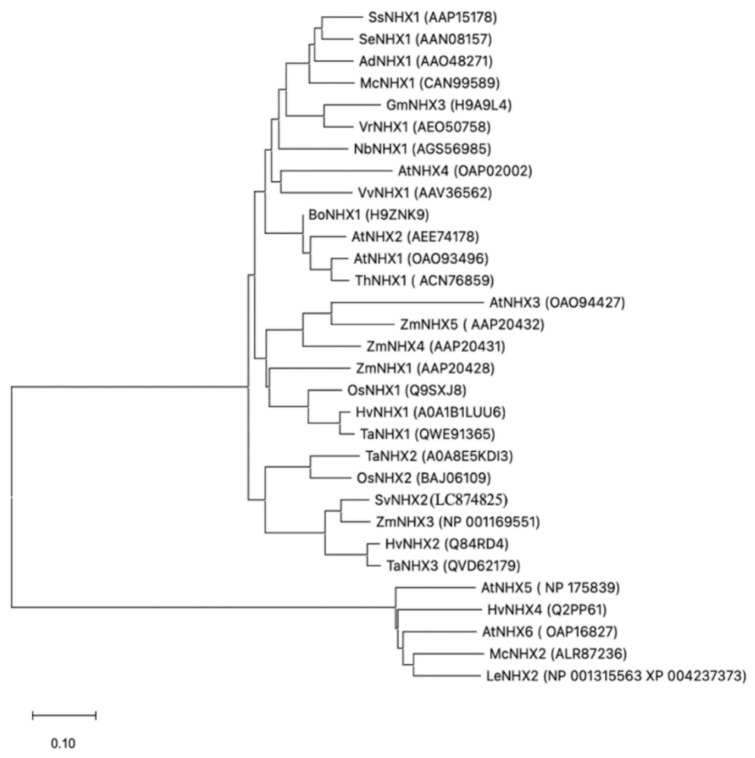
Phylogenetic analysis of NHXs. Phylogenetic analysis of NHX amino acid sequences was conducted using the MEGA11 software package. Evolutionary relationships between *SvNHX2* and NHX proteins from various monocotyledonous and dicotyledonous plants, with their accession numbers. Phylogenetic relationships were inferred using the neighbor-joining method, and the optimal tree is displayed. The evolutionary distances were calculated using the Poisson correction method and are expressed in terms of the number of amino acid substitutions per site. The scale bar represents 0.10 substitutions per site.

An NHX-like unigene was identified from *S. virginicus* RNA-Seq data [34], and the full-length *SvNHX2* gene was cloned using PCR. Phylogenetic analysis of the SvNHX2 amino acid sequence together with 30 NHX members from 15 species—including halophytes such as *Suaeda salsa*, *Salicornia europaea*, *Mesembryanthemum crystallinum*, and *Vigna radiata*—revealed that SvNHX2 is most closely related to ZmNHX3 from *Zea mays,* followed by HvNHX2 from *Hordeum vulgare* and TaNHX3 from *Triticum aestivum* (Figure 1). This close evolutionary relationship suggests that SvNHX2 may share functional characteristics with other vacuolar NHX transporters.

### 2.2. Expression of SvNHX2 in S. virginicus Under NaCl Stress

**Figure 2:**
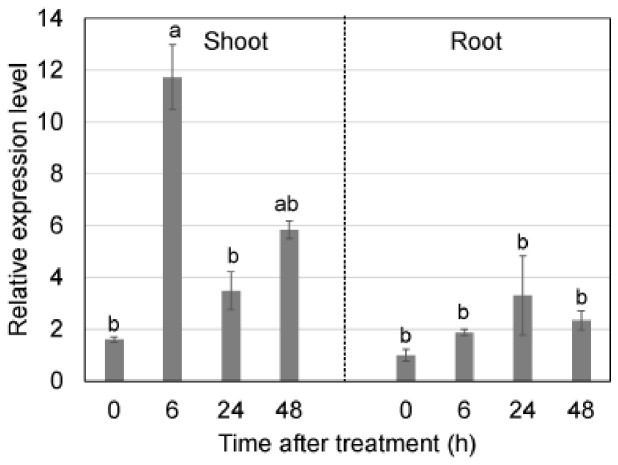
Expression profile of *SvNHX2* in *Sporobolus virginicus* under salt stress. Expression levels of *SvNHX2* at different time points after 500 mM NaCl treatment were determined by qRT-PCR. Expression levels relative to those in roots at 0 h after treatment (1.0) are shown. Data are presented as the mean ± SE (*n* = 3, biological replicates). Mean values with different letters are significantly different at α < 0.05 using Tukey’s method.

The expression profiles of *SvNHX2* in the shoots and roots of *S. virginicus* were analyzed by qRT-PCR at 0, 6, 24, and 48 h following treatment with 500 mM NaCl based on the method used in our previous study [32] (Figure 2). *SvNHX2* transcripts were detected in both shoot and root tissues. Expression levels were consistently higher in shoots than in roots. Notably, in shoots, *SvNHX2* expression increased significantly, reaching a peak of approximately 7.33-fold at 6 h after NaCl treatment, and remained upregulated up to 48 h. In contrast, in roots, *SvNHX2* expression did not show a statistically significant increase, although a gradual rise was observed, with a peak at 24 h post-treatment.

### 2.3. Generation of Transgenic Arabidopsis Overexpressing SvNHX2

**Figure 3:**
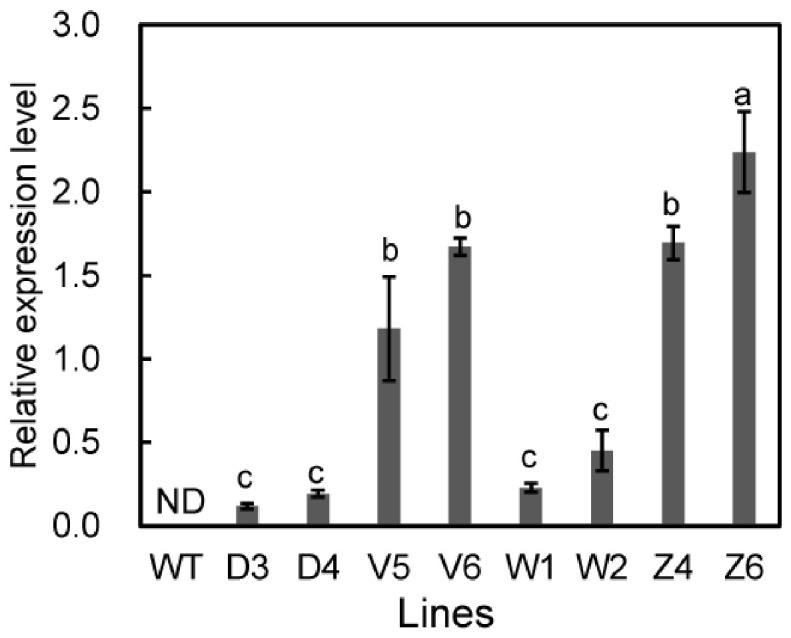
Expression levels of *SvNHX2* in transgenic *Arabidopsis* lines. Fourteen-day-old whole plants of *SvNHX2* transgenic *Arabidopsis* lines grown in 1/2 MS medium were used for RNA extraction and qRT–PCR analysis. Data are presented as the mean ± SE (*n* = 3, biological replicates). ND, Not detected. Mean values with different letters are significantly different at α < 0.05 using Tukey’s method.

The CaMV35S::SvNHX2 construct was introduced into wild-type (WT) *Arabidopsis*, and eight T_2_ lines with putative single insertions were selected based on hygromycin segregation in T_3_ progeny. While the segregation ratio alone is insufficient evidence for a single-gene insert line, we ensured that lines exhibiting abnormal segregation ratios were not used in our experiments. Expression analysis revealed variable transgene expression among the lines, with line Z6 exhibiting the highest expression, followed by lines Z4, V6, and V5 (Figure 3). Transgene expression was confirmed in all transgenic lines.

### 2.4. Salt Tolerance of Transgenic Arabidopsis

**Figure 4:**
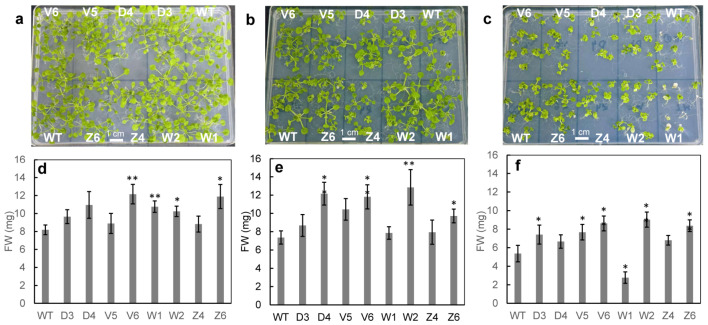
Salt tolerance test for the SvNHX2 transgenic lines. One-week-old seedlings were transplanted onto 1/2 MS agar medium supplemented with 0, 100, or 125 mM NaCl, and their fresh weight (FW) was determined after a further 2 weeks of incubation. (**a**–**c**) The appearance of transgenic lines and WT plants on 1/2 MS medium supplemented with 0, 100, or 125 mM NaCl. (**d**–**f**) Shoot FW of plants on 1/2 MS medium supplemented with 0, 100, or 125 mM NaCl. Data are presented as the mean ± SE (*n* = 8–10, biological replicates). Single and double asterisks denote significant differences compared with the values of the WT plants under the same conditions at *p* < 0.05 and *p* < 0.01, respectively, determined using Student’s *t*-test.

Our aim was to evaluate whether *SvNHX2* enhances salinity tolerance, transgenic and WT seedlings were grown on 1/2 MS medium supplemented with NaCl for 14 days, based on the method used in a similar study [25,35]. As a preliminary salt tolerance test, several transgenic lines and the WT were cultured on media supplemented with 100, 125, or 150 mM NaCl. At 100 and 125 mM NaCl, the transgenic lines exhibited better growth than the WT; however, at 150 mM NaCl, both showed severe growth inhibition and no clear difference was observed. Therefore, NaCl concentrations of 0, 100, and 125 mM were used in subsequent salt tolerance tests (Figure 4). In this experiment, because the dry weight (DW) of Arabidopsis plants was too small to measure accurately, the fresh weight (FW) was measured instead of DW. Under non-stress conditions, most transgenic lines exhibited shoot FW comparable to the WT, excluding lines V6, W1, W2, and Z6 (Figure 4a,d). In the WT, shoot FW decreased at 100 mM NaCl and was severely reduced at 125 mM NaCl (Figure 4b,c,e,f). In contrast, most transgenic lines maintained significantly higher shoot FW under both 100 mM and 125 mM NaCl, excluding lines W1 and D4 at 125 mM (Figure 4e,f). These results indicate that SvNHX2 overexpression confers salt tolerance.

**Figure 5:**
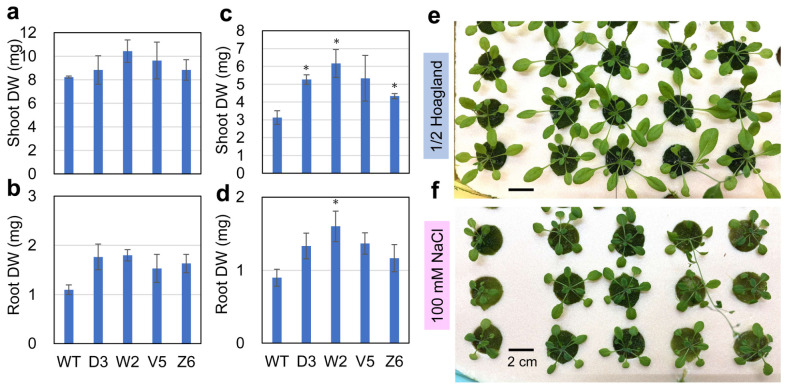
Shoot and root dry weight (DW) of the hydroponically cultured *SvNHX2* transgenic lines. Fourteen-day-old plants were transplanted to a hydroponic culture system and grown under control (0 mM NaCl) or 100 mM NaCl conditions. (**a**,**c**) Shoot DW of the plants cultivated under control and 100 mM NaCl. (**b**,**d**) Root DW of plants cultivated under control and 100 mM NaCl conditions. (**e**,**f**) The appearance of plants cultivated under control and 100 mM NaCl conditions. Data are presented as the mean ± SE (*n* = 3, biological replicates). Single asterisk denotes significant differences compared with the values in the WT plants at *p* < 0.05, determined using Student’s *t*-test.

Salt tolerance of transgenic plants was further assessed in a hydroponic system (Figure 5). Under non-stress conditions, shoot and root DW did not differ significantly between the WT and transgenic lines (Figure 5a,b,e). Under 100 mM NaCl, most transgenic lines exhibited significantly higher shoot DW compared with the WT (Figure 5c,f). Root DW of line W2 was significantly greater than that of the WT, and other transgenic lines also exhibited higher root DW than the WT (Figure 5d). These findings provide further evidence of the role of SvNHX2 in improving plant performance under salinity stress.

### 2.5. Ion Content in Transgenic Arabidopsis

**Figure 6:**
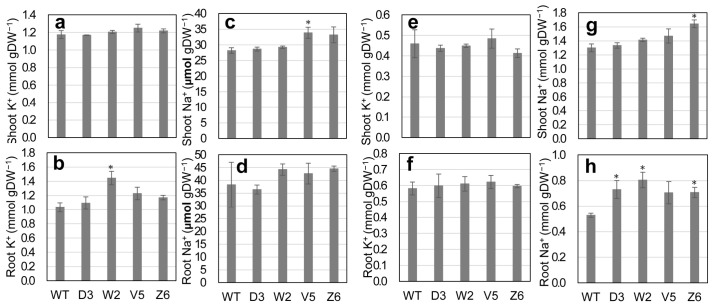
Na^+^ and K^+^ contents of shoots and roots in the *SvNHX2* transgenic lines hydroponically cultured under 0 mM NaCl or 100 mM NaCl conditions. (**a**,**b**) Shoot and root K^+^ contents under control conditions. (**c**,**d**) Shoot and root Na^+^ contents under control conditions. (**e**,**f**) Shoot and root K^+^ contents under 100 mM NaCl conditions. (**g**,**h**) Shoot and root Na^+^ contents under 100 mM NaCl conditions. The data are presented as the means ± SD (*n* = 3, biological replicates). Single asterisk denotes significant differences compared to the WT plants at *p* < 0.05, determined using Student’s *t*-test.

To determine the effect of SvNHX2 overexpression on ion accumulation, K^+^ and Na^+^ contents were measured in shoots and roots of hydroponically grown plants subjected to 0 or 100 mM NaCl (Figure 6). Under non-stress conditions, both K^+^ and Na^+^ contents were similar between the WT and transgenic lines in both organs (Figure 6a–d), excluding the shoot Na^+^ content of line V5. Under 100 mM NaCl, root Na^+^ contents in most transgenic lines and shoot Na^+^ contents in line Z6 were significantly higher than those in the WT (Figure 6g,h). In contrast, shoot and root K^+^ contents remained comparable to those in the WT (Figure 6e,f). These results indicate that *SvNHX2* overexpression promotes Na^+^ accumulation, particularly in roots.

## 3. Discussion

We identified the *SvNHX2* gene from the halophyte *S. virginicus* and demonstrated its salt-responsive expression in both shoots and roots, with stronger induction in shoots (Figure 2). This result suggests that SvNHX2 may primarily play a role in shoots rather than roots. The rapid increase in *SvNHX2* expression at 6 h after NaCl treatment and the subsequent decrease at 24 and 48 h suggest that this protein may play a more important role in early osmoregulation. This expression pattern is consistent with *NHX* genes in rice [36], tomato [37], cotton [38], and barley [39]. In rice and barley, salt-tolerant varieties exhibited higher *NHX* expression than sensitive varieties, supporting their important role in salt tolerance. NHX transporters function primarily by sequestering Na^+^ into vacuoles, thereby reducing cytosolic Na^+^ toxicity [6].

Phylogenetic analysis revealed that *SvNHX2* is closely related to *ZmNHX3* from maize (Figure 1). The results of previous studies have demonstrated that *ZmNHX3* enhances salt tolerance by promoting vacuolar Na^+^ sequestration [40,41], suggesting that *SvNHX2* may play a similar role.

Although no clear correlation was observed between transgene expression levels and salt tolerance, *SvNHX2*-overexpressing *Arabidopsis* lines exhibited significantly improved FW and DW under salinity stress (Figure 4 and Figure 5). Although we did not confirm the expression of the SvNHX2 protein or its localization to the vacuolar membrane in this study, the phenotype of these transgenic lines suggests that this protein is expressed and functions in the vacuolar membrane. The cultivation conditions used in this study (agar medium and hydroponics) differ from soil conditions in that they do not involve soil-buffering capacity or osmotic gradient changes. Therefore, it is thought that the hydroponic system imposes stronger NaCl stress on plants compared to soil cultivation. Conversely, in the case of agar medium, plants grow in a Petri dish under high humidity. It is therefore possible that the amount of water transpiration from the leaves is suppressed, thus potentially mitigating salt stress. To perform a more comprehensive evaluation of the salt tolerance of the transgenic lines, they should be evaluated by cultivating in soil. Our results are in line with reports on overexpressing *NHX* genes from glycophytes, such as *AtNHX1*, *TaNHX2*, *OsNHX2*, *PvNHX1*, *StNHX*, and *LeNHX2* [6,10,11,12,13,14,15,16,17,18]. These transgenic plants exhibited better growth under 50–200 mM NaCl stress compared to the WT plants. Halophytes generally exhibit stronger or constitutive NHX activity compared with glycophytes [42,43,44], suggesting a potentially higher Na^+^ sequestration capacity. Overexpression of halophytic *NHXs,* such as *ThNHX1* [20], *SsNHX1* [21], *SsNHX1* [22], *HcNHX1* [23], *SeNHX1* [24,25], *BvNHX1* [27], and *PutNHX1* [25], also enhances salt tolerance in glycophytic plants; however, their effects were generally comparable to those of glycophytic *NHXs*. Although *SsNHX1*-overexpressing alfalfa [21] and *SucNHX1*-overexpressing *Arabidopsis* [26] reportedly tolerated up to 400 mM and 300 mM NaCl, respectively, these experiments were conducted in soil, where salt stress is mitigated by the soil-buffering capacity compared with hydroponic culture [32]. In this study, *SvNHX2*, derived from *S. virginicus*, which exhibits remarkably high salt tolerance, conferred salt tolerance comparable to that of other halophytic *NHXs*, in addition to that of glycophytic *NHXs*. Overexpression of *ZxNHX1* from the halophyte *Zygophyllum xanthoxylum* was reported to result in greater growth enhancement than *AtNHX1*-overexpressing plants under 50 and 200 mM NaCl, although the differences were negligible [45]. Collectively, these findings suggest that NHX function in halophytes is not superior to that of glycophytes. Because salt tolerance is a complex multigenic trait exhibiting heterosis, dominance and additive effects, no individual trait will necessarily correlate with overall plant performance [9], even if it originates from halophytes. The authors of future studies could explore the possibility of enhancing salt tolerance by overexpressing multiple genes related to the regulation of Na^+^ and K^+^ derived from halophytes.

Ion analysis results demonstrated that *SvNHX2* overexpression increased Na^+^ accumulation in roots under salt stress (Figure 6), suggesting that SvNHX2 preferentially mediates Na^+^/H^+^ exchange to enhance vacuolar Na^+^ sequestration. Vacuolar Na^+^ compartmentalization is a crucial mechanism, not only for mitigating Na^+^ toxicity in the cytosol but also for contributing to osmotic adjustment under salinity stress [46,47]. Several transgenic lines exhibited higher shoot FW and increased shoot Na^+^ contents even under non-stress conditions (Figure 4d and Figure 6c). These results suggest that enhanced Na^+^ compartmentalization has a positive effect on plant growth even under non-stress conditions. Increased root Na^+^ accumulation under salt stress has been reported in numerous studies on NHX-overexpressing lines, including *AtNHX1* [10,12], *TaNHX2* [13,14], and *StNHX1* [17] from glycophytes and *SsNHX1* [21], *SsNHX1* [22], and *BvNHX1* [27] from halophytes. An exception is *PvNHX1*, which exhibited decreased root Na^+^ accumulation under salt stress [16]. In contrast to these studies, shoot K^+^ content in *SvNHX2* transgenic lines remained similar to that of the WT under salt stress. Most *NHX*-overexpressing lines in the above-mentioned studies exhibited elevated K^+^ contents under salt stress, suggesting a preference for K^+^/H^+^ over Na^+^/H^+^ exchange. Further studies on tonoplast ion fluxes will be necessary to clarify the substrate selectivity of SvNHX2. Overall, our findings suggest that SvNHX2 may sequester harmful Na^+^ in vacuoles and enhance water absorption in transgenic cells, particularly root cells, by utilizing Na^+^ as an osmolyte.

## 4. Materials and Methods

### 4.1. Production of Transgenic Arabidopsis Plants

*NHX* gene homologs were identified from previously constructed unigene datasets assembled from RNA-Seq data of *S. virginicus* [34]. Among the identified *NHX-like* unigenes, a sequence designated *SvNHX2* (DNA Data Bank of Japan, accession no. LC874825) was amplified by means of polymerase chain reaction (PCR) using gene-specific primers SvNHX2F1 (5′-CACCATGGGGCTAGATTGGGGAGGCCTT-3′) and SvNHX2R2 (5′-TGTGGTTCAAGTATGCTCTGCCTC-3′). The amplified fragment was cloned into the pENTR vector (Thermo Fisher Scientific, Tokyo, Japan) to generate the entry construct pENTR-SvNHX2. An LR recombination reaction (Gateway cloning system; Thermo Fisher Scientific) was performed between pENTR-SvNHX2 and the destination vector pGH1 [48] to generate the expression construct pGH1-SvNHX2, in which *SvNHX2* was driven by the *CaMV35S* promoter. Wild-type (WT) *Arabidopsis thaliana* (ecotype Columbia-0) plants were transformed with pGH1-SvNHX2 using the floral dip method with *Agrobacterium tumefaciens* strain GV3101.

### 4.2. Phylogenetic Analysis of NHX Protein

Multiple sequence alignments of NHX amino acid sequences from monocotyledonous and dicotyledonous species, including halophytes, were performed using ClustalW. Phylogenetic analysis was conducted using MEGA11 software [49]. A phylogenetic tree was constructed using the Jones–Taylor–Thornton substitution model and the neighbor-joining method.

### 4.3. Plant Growth Conditions and Salt Stress Treatments

Transgenic and WT *Arabidopsis* plants were grown on half-strength Murashige and Skoog (1/2 MS) medium supplemented with 1% (*w*/*v*) sucrose and 0.8% (*w*/*v*) agar (pH 5.7). Plants were incubated at 23 °C under a 16 h light/8 h dark photoperiod with a light intensity of approximately 60 µmol m^−2^ s^−1^.

### 4.4. Seedling-Stage Salt Tolerance Assay

Seven-day-old seedlings were transferred to 1/2 MS medium supplemented with 1% sucrose and 0, 100, or 125 mM NaCl (pH 5.7). After 14 days of treatment, shoots were harvested, and FW was measured. Data are presented as the mean ± SE (*n* = 8–10, biological replicates).

### 4.5. Vegetative-Stage Salt Tolerance Assay (Hydroponic Culture)

For vegetative-stage analysis, plants were grown hydroponically using the Home Hyponica Karen system (Kyowa Co., Ltd., Osaka, Japan) filled with 1/2 strength Hoagland solution supplemented with 0.2% MES buffer, as described previously [50]. Fourteen-day-old seedlings grown on 1/2 MS agar medium were transferred to the hydroponic system. Salt stress was applied by adding 50 mM NaCl to the nutrient solution on days 7 and 9 after transfer, resulting in a final concentration of 100 mM NaCl. The imposition of abrupt NaCl stress causes detrimental damage to plants, and this effect is particularly pronounced in hydroponic culture systems, which lack the buffering capacity of soil. Therefore, we applied this two-step salt stress treatment. Shoots and roots were harvested separately on day 14 after transfer, rinsed with distilled water, blotted dry, and weighed to determine FW. Dry weight (DW) was measured after drying the tissues at 60 °C overnight. Data are presented as the mean ± SE (*n* = 3, biological replicates).

### 4.6. Measurement of Ion Content

Dried plant tissues were ground into a powder using a crusher mill (Tokken, Noda, Japan). Deionized water (0.5 mL) was added to each sample, and soluble ions were extracted by incubation at 60 °C overnight. Na^+^ and K^+^ concentrations in the extracts were measured using a LAQUAtwin ion meter (Horiba, Tokyo, Japan). Data are presented as the mean ± SE (*n* = 3, biological replicates).

### 4.7. Quantitative RT-PCR

To examine the salt responsiveness of *SvNHX2* in *S. virginicus*, plants were hydroponically cultured in 1/2 MS solution as previously described [34] and then transferred to medium containing 500 mM NaCl. Samples were collected for RNA isolation at 0, 6, 24, and 48 h after treatment.

To determine *SvNHX2* expression levels in transgenic *Arabidopsis*, 14-day-old whole seedlings (*n* = 3 biological replicates) were harvested for RNA extraction. The RNAiso Plus (Takara Bio, Otsu, Japan) was used to extract the total RNA. First-strand cDNA was synthesized from 250 ng of total RNA using the QuantiTect Reverse Transcription Kit (Qiagen, Hilden, Germany). Quantitative RT-PCR was performed as previously described [48]. *SvNHX2* transcript levels were quantified using the primers SvNHX2-F6 (5′-GCTTGGTTGTGATGATGTGGTGTG-3′) and SvNHX2-R3 (5′-ACTTGAAACCCGGCATTGAAGATG-3′). Relative gene expression levels were calculated using the ΔΔCt method. *Actin* was used as the reference gene for *S. virginicus*, and *ubiquitin 5* (*Ubi5*, AT3G62250.1) was used for *Arabidopsis*. Data are presented as the mean ± SE (*n* = 3, biological replicates).

### 4.8. Statistical Analysis

Statistical analyses were performed using Student’s *t*-test for pairwise comparisons or using Tukey’s multiple contrasts method for multiple comparisons. In Student’s *t*-test, differences were considered statistically significant at *p* < 0.05 or *p* < 0.01. In Tukey’s multiple contrasts method, different letters above bars indicate significant differences among genotypes at α = 0.05.

## 5. Conclusions

Transgenic *Arabidopsis* plants overexpressing *SvNHX2* exhibit enhanced growth under salt stress. This improvement is associated with increased root Na^+^ accumulation, highlighting the role of SvNHX2 in vacuolar Na^+^ sequestration and salt tolerance. Our results indicated that the halophytic *SvNHX2* conferred salt tolerance to *Arabidopsis* at levels similar to glycophytic *NHXs* and could serve as a potential gene for engineering salt-tolerant crops.

## Data Availability

The datasets generated and/or analyzed in the present study are available from the corresponding author on reasonable request.

## Abbreviations

The following abbreviations are used in this manuscript:

WT	Wild type
FW	Fresh Weight
DW	Dry Weight
MS	Murashige and Skoog
CaMV35S	Cauliflower mosaic virus 35S
qRT-PCR	quantitative reverse transcription-PCR
NHX	Na^+^/H^+^ antiporter
